# Repurposing of Antibiotic Sulfisoxazole Inhibits Lipolysis in Pre-Clinical Model of Cancer-Associated Cachexia

**DOI:** 10.3390/biology10080700

**Published:** 2021-07-22

**Authors:** Sai V. Chitti, Akbar L. Marzan, Sanjay Shahi, Taeyoung Kang, Pamali Fonseka, Suresh Mathivanan

**Affiliations:** Department of Biochemistry and Genetics, La Trobe Institute for Molecular Science, La Trobe University, Melbourne, VIC 3086, Australia; 19573709@students.latrobe.edu.au (S.V.C.); 19572414@students.latrobe.edu.au (A.L.M.); 18604885@students.latrobe.edu.au (S.S.); 19174837@students.latrobe.edu.au (T.K.); P.Fonseka@latrobe.edu.au (P.F.)

**Keywords:** cancer-associated cachexia, C26 colon carcinoma, sulfisoxazole, white adipose tissue, lipolysis

## Abstract

**Simple Summary:**

A large number of advanced cancer patients suffer from a gradual weight loss manifested as muscle and fat loss. This condition is known as cancer-associated cachexia. Cachexia limits tolerance of anti-cancer treatments, surgeries, and quality of life of individual who have cancer. Unfortunately, there are no potential agents available to treat cancer-induced cachexia. Here, we investigated the efficacy of the drug Sulfisoxazole in both cell and animal-based experiments. Our results demonstrated that upon treatment with Sulfisoxazole there was partial reduction of fat loss and improved overall well-being of the mice with cancer cachexia. Furthermore, cell-based analysis revealed that Sulfisoxazole reduces the loss of fat cells size and number. Thus, oral administration of Sulfisoxazole could be a promising means for the treatment of cancer-associated cachexia if it is combined with agents that could potentially inhibit muscle loss and/or anti-cancer agents.

**Abstract:**

Clinical management of cancer-associated cachexia, a multi-organ wasting syndrome, has been challenging without effective treatment strategies. An effective treatment that directly targets cancer-induced wasting is desperately needed to improve the quality of life and the survival of cancer patients. Recently, an antibiotic SFX was shown to have anti-tumour and anti-metastatic effects in mouse models of breast cancer. Hence, in this study, we examined the efficacy of SFX in the treatment of cancer-induced cachexia. C26 cachexic mice models were administered with SFX, and the tumour volume and body weight were regularly measured. Blood glucose, skeletal muscles, and adipose tissue were examined at the endpoint. Contrary to a previous study, SFX did not reduce the tumour volume in mice bearing C26 cells. Administration of SFX neither revealed any survival benefit nor rescued C26 cachectic mice from muscle wasting. Interestingly, SFX administration partially rescued (~10%) tumour-induced weight loss by preserving both the subcutaneous and intestinal fat mass. Together, these results suggest that the administration of SFX could partially rescue cancer-induced weight loss by inhibiting lipolysis. As anti-cachexia therapies are scarce, the results could facilitate the design of combinatorial therapies involving SFX, standard-of-care chemotherapeutics, and drugs that inhibit muscle atrophy for the treatment of cancer cachexia.

## 1. Background

Cachexia is a multifactorial metabolic syndrome characterised by the dramatic loss of skeletal muscle mass, which is often accompanied by fat loss [1]. It is a devastating consequence seen in patients with acquired immune deficiency syndrome [2], burns [3], cancers [4,5], chronic obstructive pulmonary disease [6], chronic heart [7] and renal failures [8], diabetes [9], and sepsis [10,11,12]. During these disorders, the basal energy expenditure increases, which enhances the fat and protein catabolism primarily from the energy reserve organs and thus leads to wasting [8]. Along with the skeletal muscles and fat tissue, cachexia also affects major organs and tissues such as the brain, heart, liver, kidneys, bone, and gastrointestinal tract, thereby representing a multi-organ syndrome [13]. In the context of cancer, it is estimated that half of all patients are affected by cachexia [14]. The chronically increased energy imbalance during cancer-associated cachexia is attributed to both intrinsic (chronic inflammation, metabolic alterations) and extrinsic (reduced physical activity, susceptibility to infections, fatigue, anorexia) properties [14,15,16].

Due to the multifactorial pathogenesis of cancer-associated cachexia, the progress in understanding the mechanism and translating the knowledge to clinical therapy has been limited [17]. It has been established that numerous mediators such as cytokines, adipokines, myokines, tumour-derived factors contribute and or are associated with the aetiology of the disease by inducing lipolysis, protein degradation, along with the inhibition of protein and lipid biosynthetic pathways [18,19,20]. However, several clinical trials involving anti-cachectic factors have reported minimal to no clinical benefit [21,22,23,24]. Furthermore, clinical trials have demonstrated that cachexia cannot be reversed with exercise and nutritional support [25,26]. As a consequence, there are no agents approved by the Food and Drug Administration (FDA) for the treatment of cancer-associated cachexia at the moment [27,28]. Hence, there is an urgent need to identify drugs that can be used to treat cancer-associated cachexia. Among the various ways to identify a drug with clinical benefit, repurposing offers several advantages, including its prior approval for safety [29]. Recently, repurposing of an FDA-approved oral antibiotic, sulfisoxazole (SFX), in pre-clinical breast cancer models demonstrated its anti-tumour and anti-metastatic effects [30]. It has been proposed that SFX treatment reduces the release of extracellular vesicles, small membranous vesicles released by cells [31,32], and thereby reduces metastasis of these cancer cells. However, another study failed to confirm this finding that SFX reduces the release of extracellular vesicles by cancer cells [33]. Nevertheless, SFX has been used in Phase 2 clinical trials for breast cancer treatment. However, the efficacy of SFX in cancer-associated cachexia has not been investigated.

In this study, we used SFX for the treatment of cancer-associated cachexia in pre-clinical models for the first time. We were able to confirm that tumour-induced loss of fat and skeletal muscle mass is consistent with the development of cachexia using C26 tumour-bearing mice. Though SFX administration did not reduce the loss of skeletal muscle mass, it partially rescued the tumour-induced weight loss by preserving both the subcutaneous and intestinal fat mass. Importantly, this newly identified potential of SFX in reducing fat loss could facilitate new combinatorial therapies that would specifically inhibit muscle atrophy and would therefore concomitantly target both lipid and protein loss in cancer cachexia patients.

## 2. Methods

### 2.1. Cell Culture

C26 and 3T3.L1 cells were gifted by Prof. Nicholas J. Hoogenraad (La Trobe University, Melbourne, Australia). C26 cells were grown in RPMI1640 (Gibco, Life Technologies, Carlsbad, CA, USA) supplemented with 10% (*v*/*v*) fetal calf serum (FCS), 2 mM glutamine, 25 Mm HEPES, and 100 Unit/mL of penicillin-streptomycin (Gibco, Life Technologies) at 37 °C, 5% CO_2_, and 95% humidity.

3T3.L1 mouse pre-adipocytes cells were grown in DMEM, 10% (*v*/*v*) FCS and 1% (*v*/*v*) penicillin-streptomycin growth medium. To initiate differentiation, cells were cultured until confluent and replaced with differentiation medium (DMEM/F12 with 10% FCS, 1.5 µg/mL insulin, 1 µM dexamethasone, 500 µM IBMX, and 1 µM rosiglitazone) for 72 h. The differentiation medium was replaced with adipocyte maintenance medium (DMEM/F12 with 10% FCS and 1 µg/mL insulin) and replaced every 48 h. Lipid droplet accumulation was visible by light microscopy 7–10 days after the addition of the differentiation medium.

### 2.2. Chemicals and Materials

Sulfisoxazole (SFX) was purchased from MedChemExpress (cat# HY-B0323, Middlesex, NJ, USA), corn oil was purchased from Sigma-Aldrich (cat# C8267, St. Louis, MO, USA), glucometer from Aviva Accu-Chek (Roche, Germany), 3T3-L1 Differentiation Kit from BioVision (cat# K579, Milpitas, CA, USA), and Oil Red O from Sigma-Aldrich (cat# O0625, St. Louis, MO, USA). TNF-α was purchased from Abcam (cat# ab9642, Cambridge, UK).

### 2.3. CD2F1 Mice Model for Cancer-Associated Cachexia

Animal experiments were conducted in accordance with the Australian code of practice for the care and use of animals for scientific purposes and in compliance with La Trobe Ethics Committee guidelines. Blood collection, tissue sample procurement, handling, processing, and storage of samples were according to the AEC-approved ethics application (AEC18052). C26 cells (1 × 10^6^) were injected subcutaneously to the upper left flank region of 16-week-old female CD2F1 mice (purchased from Animal Resources Centre, Murdoch WA, Australia). The control group consisted of mice that did not receive cancer cells or SFX or vehicle. For pair feeding, the food intake of mice bearing C26 tumour group for 24 h was monitored, and the pair-fed group was provided with the same amount of food for the next 24 h. Tumour size and body weight were monitored daily. Tumour size was measured using a digital caliper, and the cumulative tumour volume was calculated as follows: $tumor\;volume\;\left({\mathrm{mm}}^{3}\right)=\frac{Length\times Width^{2}}{2}$. At 50 mm^3^ of tumour volume, a single dose of SFX suspended in 200 µL of corn oil was orally gavaged to CD2F1 mice daily at a concentration of 300 mg/Kg. Scientific endpoints were determined by monitoring the body conditions of the mice, such as hunching posture, signs of pain, or when the cumulative primary tumour volume reached 1500 mm^3^ or when the mice lost ≥20% of their body weight. Mice were euthanised using 5% CO_2_ asphyxiation.

### 2.4. Murine Blood Glucose Measurements

Blood collected by cardiac puncture was checked with an Aviva Accu-Chek glucometer to assess the blood glucose levels.

### 2.5. Oil Red O Staining

Cells were washed twice with 1× phosphate-buffered saline (PBS), fixed in 10% (*v*/*v*) formalin for 1 h at room temperature, and then washed twice with 1× PBS 60% (*v*/*v*) isopropanol was added to cells for 5 min and aspirated. Cells were stained with Oil Red O working solution (3 parts of 0.3% (*w*/*v*) Oil Red O dye in 2 parts of deionised water) for 5 min. Cells were rinsed with tap water until the water runs clear and viewed under a phase contrast microscope.

### 2.6. Analysis of Oil Red O Staining

Oil Red O staining analysis was carried out using automated script in Image J software (1.53c). The Oil Red O stain was colour thresholded, and a binary image was created to which a watershed filter was applied. The size of the lipid droplet was then measured by analysing particle function.

## 3. Results

### 3.1. SFX Partially Inhibits Cancer-Induced Weight Loss in C26 Tumour-Bearing Mice

**Figure 1:**
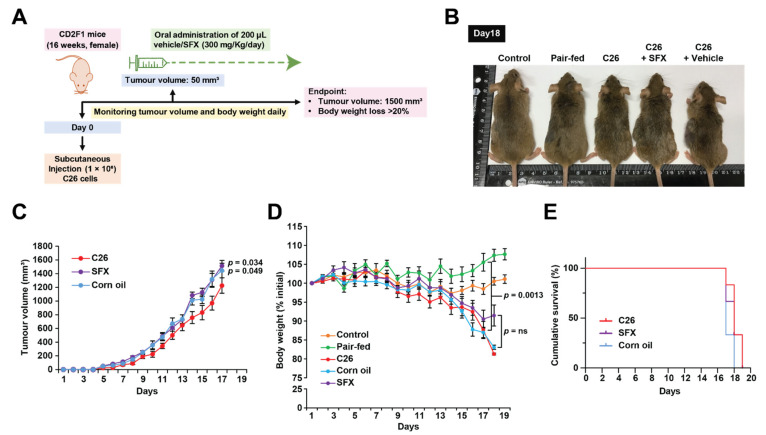
SFX partially inhibits cancer-induced weight loss in C26 tumour-bearing mice. (**A**) Schematic representation of the in vivo study. Female CD2F1 mice (16 weeks old) were injected subcutaneously with C26 (1 × 10^6^) in the upper left flank region. At 50 mm^3^ of tumour volume, a single dose of SFX suspended in 200 µL of corn oil at a concentration of 300 mg/Kg per day was orally gavaged. Tumour volume and body weight were monitored daily, and mice were sacrificed when body weight loss was ≥20% or when tumour volume reached ≥1500 mm^3^. (**B**) Representative image of the C26 bearers with SFX treatment and vehicle control upon scientific endpoint (day 18). C26 bearers and vehicle control lost ≥20% of the body weight when compared to the pair-fed and control groups. (**C**) Tumour volume was measured at the indicated time points. (**D**) Group average body weight standardised to starting weight ± SEM of 16 weeks, female CD2F1 mice. (**E**) Kaplan–Meier curve showing SFX treatment did not improve the survival of mice. All data are representing mean ± SEM, NS (not significant) unpaired two-tailed Student’s *t*-test (*n* = 6 mice per group).

To examine whether oral administration of SFX could inhibit tumour growth and cancer-induced weight loss, CD2F1 mice were inoculated with C26 tumour cells subcutaneously. Upon reaching 50 mm^3^ of tumour volume, a single dose of SFX suspended in 200 µL of corn oil or an equal volume of vehicle control (corn oil) was orally gavaged to CD2F1 mice daily at a concentration of 300 mg/Kg (Figure 1A). As both male and female mice were able to tolerate up to 300 mg/Kg of SFX for 28 days without any subacute toxicity and body weight loss [30], the concentration was chosen for the in vivo studies. By day 18 post-inoculation, mice bearing C26 tumour alone and C26 tumour with vehicle control (corn oil) groups displayed rapid weight loss, dishevelled fur, and hunched posture with a body condition score (BCS) of 1 (Figure 1B). As weight loss in tumour-bearing mice can also be attributed to anorexia, we used a pair-fed control without any tumour. The pair-fed mice group received the exact amount of food as consumed by C26 tumour-bearing mice 24 h earlier. Since the control pair-fed mice did not exhibit any significant weight loss, the possibility of anorexia was eliminated. Next, the effect of SFX on the tumour burden was examined. Contrary to a previous report that SFX decreases the tumour burden in breast cancer models [30], SFX treatment did not reduce the tumour burden of mice bearing C26 cells (Figure 1C). Corn oil by itself seemed to increase the tumour volume significantly in mice bearing C26 cells. Consistent with the literature, C26 cells induced ≥20% of weight loss in mice around day 18. However, the SFX-treated group exhibited no significant protection from cancer-associated weight loss, though a slight stabilisation in body weight was observed on day 18 (Figure 1B). Though the SFX-treated mice appeared to be hunched, they exhibited less dishevelled fur with BCS equal to 2+ (Figure 1D) compared to C26 and C26 with vehicle control groups. Hence, it was apparent that the antibiotic SFX partially reduced tumour-induced weight loss (~10%). On day 18, the corn oil group had lost an average of 18% body weight, while the SFX group exhibited an 8.5% loss in body weight. Though this ~10% change in the body weight at day 18 was observed, there was no increase in the overall survival rate (Figure 1E) of mice receiving SFX treatment. As the tumour volume was higher in the SFX-treated group (>1500 mm^3^ ethical endpoint), the mice were sacrificed despite reduced body weight loss and better BCS. To further understand this partial rescue of weight loss, the skeletal muscle and adipose tissue were examined to understand whether SFX had any benefit in preventing wasting.

### 3.2. SFX Rescues Lipolytic Loss in C26 Tumour-Bearing Mice

**Figure 2:**
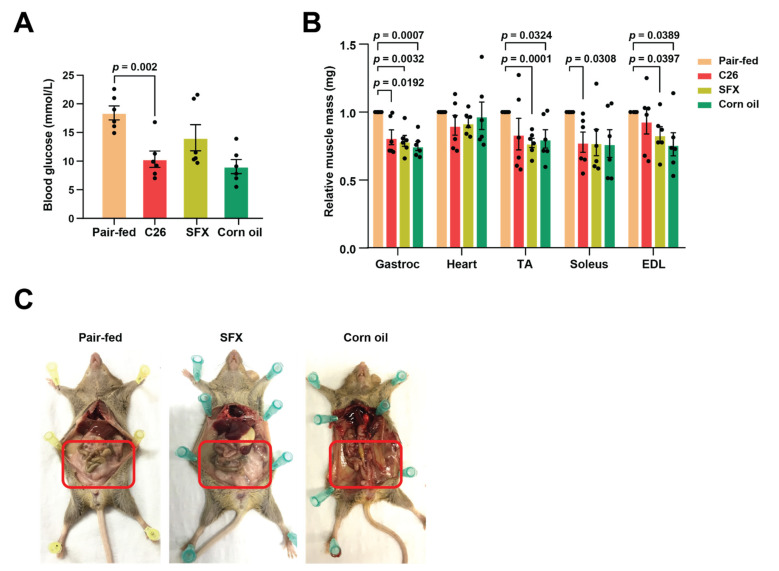
SFX rescues lipolytic weight loss in C26 tumour-bearing mice. (**A**) Dot plot representing the terminal blood glucose levels analysed using a glucometer. (**B**) Dot plot showing relative muscle mass of TA: Tibialis anterior, EDL: Extensor digitorum longus, Gastroc: Gastrocnemius, soleus, and heart, excised and weighed upon scientific endpoint. (**C**) Representative images displaying fat deposits that were retained with SFX treatment when compared to the vehicle (corn oil) treatment. Highlighted area indicating the fat depot in pair-fed and SFX-treated mice. All data are representing mean ± SEM, unpaired two-tailed Student’s *t*-test (*n* = 6 mice per group).

Although cancer-associated cachexia is a multi-organ syndrome, the weight loss is largely due to the depletion of adipose tissue and skeletal muscle mass [13,34]. Recent evidence suggests that fat loss occurs prior to the tipping of metabolic pathways to catabolism in skeletal muscles and various other organs [35,36,37]. Levels of glucose in the blood may be an indicator as in healthy individuals, lipid mobilisation is suppressed in the presence of glucose and thus results in maintenance of fat stores. Consistent with this notion, C26 mice had significantly lower levels of blood glucose (Figure 2A). However, SFX administration did not significantly increase the blood glucose levels, though levels were higher, compared to the vehicle control-treated tumour-bearing mice that had similar tumour volumes.

To understand the effect of SFX on muscle loss, selected muscle types were excised and weighed upon sacrificing mice on reaching their scientific endpoint. The mass of tibialis anterior (TA), extensor digitorum longus (EDL), gastrocnemius (gastroc), soleus (sol), and heart muscle did not show any significant difference in weight between the groups. Compared to the control group, C26 tumour-bearing mice exhibited significant loss of gastroc, TA, soleus, and EDL muscle (Figure 2B). However, SFX did not exhibit any protection from muscle loss as no significant difference in muscle mass between the C26- and SFX-treated groups was observed. Interestingly, the SFX-treated mice displayed an intact subcutaneous and intestinal adipose tissue when compared with the vehicle-treated group (Figure 2C). These results demonstrated that the cachexia seen in C26 bearers was caused primarily by the loss of adipose tissue and skeletal muscle, as established by several studies. However, SFX was only able to rescue the fat loss and not skeletal muscle wasting in C26 tumour-bearing mice. Though fat loss is proposed to be the first step in cancer-induced cachexia prior to protein catabolism in organs, SFX was able to protect these mice from adipose tissue wasting. These data also suggest that protein catabolism and fat loss may happen in tandem in cancer-associated cachexia.

### 3.3. SFX Reduces Lipolysis Mediated by C26 Conditioned Media In Vitro

**Figure 3:**
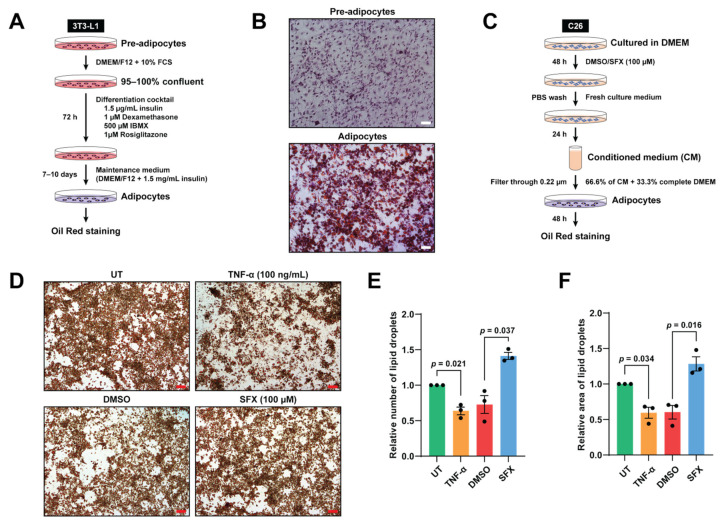
SFX reduces the loss of lipid droplets induced by C26 conditioned medium (CM) in vitro. (**A**) Schematic representation of the differentiation of pre-adipocytes to adipocytes using differentiation and maintenance medium. (**B**) Cells that were fully differentiated into adipocytes were stained with Oil Red O. First column: undifferentiated pre-adipocytes. Second column: differentiated adipocytes showing lipid droplets under a phase contrast microscope (purple staining represents hematoxylin). Magnification of 10×, scale bar: 200 µm. (**C**) Schematic representation of the cell culture, drug treatment and collection of CM. C26 cancer cells were cultured in DMEM medium with SFX (100 µM) or vehicle alone for 48 h. Following PBS wash, C26 cancer cells were incubated in DMEM complete medium for 24 h to produce CM. (**D**) Oil Red O lipid staining under a phase contrast microscope upon incubation with DMEM complete medium (untreated), 100 ng/mL TNF-α (positive control), C26 SFX, or vehicle alone treated C26 CM treatment for 48 h. Magnification of 10×, scale bar: 200 µm. (**E**,**F**) Dot plots representing quantification of the relative total number and area of lipid droplets, respectively, using ImageJ. All data are representing mean ± SEM. Statistical significance was examined by paired two-tailed Student’s *t*-test, *n* = 3 biologically independent experiments.

In order to examine the effect of SFX treatment on lipolysis in vitro, we first differentiated 3T3-L1 pre-adipocytes to mature adipocytes using a differentiation and maintenance medium (Figure 3A). As shown by Oil Red O, 3T3-L1 cells treated with differentiation and maintenance medium acquired the characteristics of mature adipocytes such as intracytoplasmic lipid accumulation, showing typical white adipocyte morphology (Figure 3B). Next, to demonstrate lipolysis in vitro, adipocytes were incubated with TNF-α, which has been previously shown to induce lipolysis (Figure 3D). To examine whether SFX treatment can reduce lipolysis, adipocytes were incubated with 66.6% of C26 conditioned medium (CM) collected after 24 h of SFX or vehicle alone treatments. Consequentially, SFX-treated C26 CM significantly decreased the loss of lipid droplets and improved the percentage area of lipid droplets compared to the vehicle alone treatment, which is evident from the Oil Red O staining (Figure 3D–F). Consistent with our in vivo findings, this result demonstrates the SFX inhibits lipolysis and supports the sparing of fat mass upon SFX treatment induced during cancer-associated wasting.

## 4. Discussion

Poor understanding of the signalling pathways that drive cachexia and the multifactorial nature of the disease have limited the discoveries of effective treatment options. Though several molecules have been associated with cancer cachexia, therapies targeting these molecules or appetite stimulants have been shown to be ineffective in patients in the context of muscle wasting and fat loss. Fat constitutes 80–90% of the body’s reserved fuel, and whole-body fat loss is a foremost feature of cancer-induced wasting observed during advanced stages of aggressive cancers [38]. Colon cancer is one such aggressive cancer, where approximately 48–60% of the patients die due to cachexia [39]. Hence, to identify drugs that may have clinical benefits in the treatment of cancer-associated cachexia, we sought out to examine FDA-approved drugs that are repurposed for cancer therapy. Recently, it has been proposed that the antibiotic SFX exhibited anti-cancer and anti-metastatic effects in breast cancer models [30].

As in vitro models of cancer cachexia that measure atrophy are not robust, pre-clinical models using C26 cachexia mice were used in this study. For the first time, the efficacy of SFX, an FDA-approved antibiotic in Phase II clinical trials for breast cancer treatment, in treating cancer-associated cachexia was examined. In this study, we report that (1) SFX treatment had no significant effect on reducing C26 tumour growth; (2) SFX reduces lipolysis and partially prevents C26 tumour-induced weight loss in mice. SFX treatment slowed down the overall weight loss by approximately 10% (day 18) and improved the BCS of the mice; and (3) SFX treatment reduces 3T3-L1 adipocytes lipolysis caused by C26 cell condition medium in vitro. Thus, oral administration of SFX could be a promising means for the treatment of cancer-associated cachexia if it is combined with agents that could potentially inhibit muscle atrophy and/or tumour burden. At present, drugs for the treatment of cancer-induced muscle atrophy include natural compounds such as eicosapentaenoic acid [40,41], resveratrol [42,43], ghrelin [44], enzyme inhibitors such as Cox2 inhibitors such as celecoxib, meloxicam [45,46,47], and phosphodiesterase inhibitors such as torbafylline, pentoxifylline [48,49], beta-adrenergic receptor agonists (formoterol) [50,51], and anti-cytokine agents, which include anti-TNFα, thalidomide [52,53,54]. Hence, SFX can be tested with these agents that preserve the muscle mass, and it is worthwhile developing a novel combinatorial regime for treating cancer cachexia.

It is established that fat and muscle percentage in mice depends on various factors such as strain, breeding strategies, age, sex, diet, and environmental conditions [55]. The overall mean weight of 40 male strains was 26.3 g (26% fat and 73% lean muscle), while the average weight of 40 female strains was 20.1 g (22.9% fat and 76% lean muscle). As we used female mice in this study, fat is expected to contribute ~23% of the body weight. The CD2F1 mice strain is a cross between Balb/c and DBA2 mice strains, and the scientific endpoint was based on body condition score (BCS), tumour volume (1500 mm^3^), and body weight loss (>20%). Hence, the 10% overall body weight benefit provided by SFX treatment could have been contributed by more than 50% of today’s fat mass preservation. Additional studies are needed to understand and quantify these fat mass changes in time and dose-dependent manner.

Although our data implicate the sparing of fat mass and improving the overall BCS upon SFX treatment, it is important to consider that various tumour types direct fat loss at different rates depending upon tumour site, mass, and stage [39]. Hence, additional studies are needed to examine the efficacy of SFX in combination with other drugs in the treatment of various tumour types. Furthermore, as the partial anti-cachectic effect was due to the restoration of fat mass, it is important to understand the mechanism that was impaired by SFX treatment to develop additional therapies to treat cancer cachexia. Furthermore, temporal analysis to understand the effect of SFX is needed as at the endpoint, the adipose tissue is completely lost in C26 and vehicle control groups, impeding any comparative analysis on these tissues. In addition, corn oil might not be the ideal vehicle control as it significantly increased the tumour volume compared to the C26 group (Figure 1C). Nevertheless, this study highlights the potential utility of SFX, an FDA-approved drug, in combination with standard-of-care chemotherapeutic drugs and inhibitors of proteolysis to treat cancer-induced wasting.

## 5. Conclusions

Overall, in this study, we used SFX for the treatment of cancer-associated cachexia in both in vitro and pre-clinical models for the first time. Though SFX administration did not reduce the loss of skeletal muscle mass, it partially rescued the tumour-induced weight loss by preserving both the subcutaneous and intestinal fat mass. As anti-cachexia therapies are scarce, the results could facilitate the design of combinatorial therapies involving SFX, standard-of-care chemotherapeutics, and drugs that inhibit muscle atrophy for the treatment of cancer cachexia.

## Data Availability

Data are available from the corresponding author upon a reasonable request.

## Abbreviations

**SFX**	Sulfisoxazole
**FDA**	Food and Drug Administration
**FCS**	Fetal Calf Serum
**BCS**	Body Condition Score
**TA**	Tibialis Anterior
**EDL**	Extensor Digitorum Longus
